# Using a Mixed Model to Explore Evaluation Criteria for Bank Supervision: A Banking Supervision Law Perspective

**DOI:** 10.1371/journal.pone.0167710

**Published:** 2016-12-19

**Authors:** Sang-Bing Tsai, Kuan-Yu Chen, Hongrui Zhao, Yu-Min Wei, Cheng-Kuang Wang, Yuxiang Zheng, Li-Chung Chang, Jiangtao Wang

**Affiliations:** 1 School of Economics & Management, Shanghai Maritime University, Shanghai, China; 2 Law School, Nankai University, Tianjin, China; 3 Zhongshan Institute, University of Electronic Science and Technology of China, Guangdong, China; 4 School of Business, Dalian University of Technology, Panjin, China; 5 Business School, Nankai University, Tianjin, China; 6 College of Tourism and Service Management, Nankai University, Tianjin, China; 7 Guangzhou Vocational College of Science and Technology, Guangzhou, China; Southwest University, CHINA

## Abstract

Financial supervision means that monetary authorities have the power to supervise and manage financial institutions according to laws. Monetary authorities have this power because of the requirements of improving financial services, protecting the rights of depositors, adapting to industrial development, ensuring financial fair trade, and maintaining stable financial order. To establish evaluation criteria for bank supervision in China, this study integrated fuzzy theory and the decision making trial and evaluation laboratory (DEMATEL) and proposes a fuzzy-DEMATEL model. First, fuzzy theory was applied to examine bank supervision criteria and analyze fuzzy semantics. Second, the fuzzy-DEMATEL model was used to calculate the degree to which financial supervision criteria mutually influenced one another and their causal relationship. Finally, an evaluation criteria model for evaluating bank and financial supervision was established.

## Introduction

Financial supervision entails that a specialized agency established by the financial management authority of a country, based on a specific working procedure, uses economic and administrative measures to supervise the financial practices of various financial institutions and to ensure the authenticity, legality, and economic efficiency of financial activities occurring in various financial institutions [1–2].

Calvo and Mendoza [3] indicated that financial fragility can induce unpredictable changes in asset markets, and thus, financial institutions can become highly indebted and risk closure. This potential danger can constantly threaten the financial institutions, and therefore, financial early-warning and monitoring systems must be introduced to maintain the stability of financial asset prices. Aldlaigan and Buttle [4] asserted that moral hazard caused excessive investment and incurred bank fragility. The government of a country is implicitly responsible for banks, and the central bank is the lender of last resort for banks. Moreover, advanced countries practice the same concept. When a consensus is formed, financial institutions are willing to take risks, thereby resulting in financial fragility and crises. Jorion [5] observed that when the economy was overheated, banks became indiscriminately optimistic and excessive credit expansion occurred. Consequently, the economic overheat became increasingly severe. When the economy was recessive, banks became irrationally pessimistic and excessive credit crunch occurred. Economic contraction and financial endogenous instability then became exceedingly severe.

Financial supervision means that monetary authorities have the power to supervise and manage financial institutions according to laws. Monetary authorities have this power because of the requirements of improving financial services, protecting the rights of depositors, adapting to industrial development, ensuring financial fair trade, and maintaining stable financial order. Hu [6] indicated that the passive function of financial supervision was to improve the operation of financial services and to maintain stable financial order. The positive function was to protect the rights of depositors and ensure fair trade for financial systems according to financial environments.

Because of financial liberalization and globalization, innovative financial activities have become prevalent, banking operations have become complex, and financial risks have increased. To manage the rapid change of financial environments effectively, all major countries endeavor to promote financial reform, review financial supervision systems and related laws and regulations, improve financial supervision methods, urge banks to enhance risk management and corporate governance, ameliorate financial systems, and enhance financial competitiveness.

Most studies investigating banking supervision indicators have assumed that the evaluation criteria are independent of each other, implying that they do not mutually influence one another and are not causally related. This assumption has limited research on banking supervision indicators. The decision-making trial and evaluation laboratory (DEMATEL) model could be used to transform a complex system into one with well-structured causal relationships by simplifying the relationships between criteria in the system into cause–effect relationships. By quantifying the interrelationships among criteria, we identified the core problems of the complex system and determined how to improve the system.

To establish evaluation criteria for bank supervision in China, this study integrated fuzzy theory and the decision making trial and evaluation laboratory (DEMATEL) and proposes a fuzzy-DEMATEL model. First, fuzzy theory was applied to examine bank supervision criteria and analyze fuzzy semantics. Second, the fuzzy-DEMATEL model was used to calculate the degree to which financial supervision criteria mutually influenced one another and their causal relationship. Finally, an evaluation criteria model for evaluating bank and financial supervision was established.

## Literature Review

### Financial supervision systems and evaluation criteria

Currently, four financial supervision approaches have been adopted internationally, namely the institutional, functional, integrated, and twin peaks approaches [7–10]. These four approaches are elaborated as follows:

1 Institutional approach

In this approach, financial institutions are supervised and managed according to their categories (e.g., banks, security firms, or insurance companies). A regulator for a financial institution defines the scope of services that the financial institution can provide and supervises the financial institution from the perspective of security. China, Mexico, and Hong Kong adopt this approach.

2 Functional approach

In the functional approach, financial institutions are supervised and regulated according to their business type. Each business type is supervised by an independent regulatory authority. By using a purely functional approach, if a financial institution undertakes various types of business (e.g., the banking and securities business), then the financial institution would be supervised and regulated by various financial regulatory authorities. Each regulatory authority regulates and manages one type of business and considers financial security matters for the specific type of business. Italy and France adopt this approach.

3 Integrated approach

In the integrated approach, a single competent authority is responsible for regulating the financial sector and supervising financial security. This approach has been receiving increasing attention in recent decades because numerous financial institutions have provided comprehensive financial services and their financial businesses cannot be categorized simply according to their operation functions. Therefore, an integrated regulatory authority is required. This model is also called the financial services authority (FSA) model. The FSA in the United Kingdom is representative of the FSA model. In addition, Germany and Taiwan adopt the integrated approach.

4 Twin peaks approach

The twin peaks approach is based on the concept of regulation by objective. This approach distinguishes two regulatory functions: regulating financial businesses and supervising financial security. In other words, business practices and system stability require separate and independent regulatory authorities (i.e., “twin peaks”). Accordingly, by using this approach, retail-type and wholesale-type financial businesses are required to meet different regulatory standards. Australia and the Netherlands adopt this approach.

In 1979, the US Federal Financial Institution Examination Council announced a Uniform Financial Institutions Rating System and recommended that various financial supervisory authorities adopt the CAMELS rating system (applying capital adequacy, asset quality, management, earnings, liquidity, and sensitivity to market risks as evaluation criteria) [7].

In 2003, the FSA in the United Kingdom began promoting a new evaluation system for bank risks. The risk evaluation model comprises four business risk groups (strategic risks; market, credit, and operational risks; financial soundness; and customer nature and product services) and six control risk groups (treatment of customers; organization; internal system and control; board of directors, management, and employees; business practices; and regulatory compliance culture) [2].

To respond to the rapid change of international financial situations, the governments of various countries effectively adjusted their supervision policies and considered the examination of financial supervisory authorities and financial early-warning systems extremely crucial. In September 1997, the Basel Committee on Banking Supervision for the Bank for International Settlements announced the Core Principles for Effective Banking Supervision to provide financial supervisory authorities in various countries with evaluation criteria for evaluating the quality of financial supervisory systems. In addition, after the global financial crisis in December 2010, the Basel Committee on Banking Supervision, announced a New Basel Capital Accord (Basel III), which has been a major reform in international banking supervision in recent years and served as a direction for formulating effective supervision practices [11].

### Systems and laws for banking supervision in China

The development of financial reform and banking supervision in China can be divided into five phases [10–13]:

1 Market-oriented planned economic period (1979–1983)

During this period, banking practices included deposits, loans, and monetary transfers and did not require assessing credit or operational risks. The governmental supervisory system focused on policy and administrative management without stipulating supervision regulations and operations such as those stipulated for a market economic system.

2 Period for financial institution legalization and foreign exchange adjustment (1984–1992)

The Industrial and Commercial Bank of China was founded in 1984 and undertook industrial and commercial credit business originally operated by the People’s Bank of China. Subsequently, the Industrial and Commercial Bank of China, China Construction Bank, Agricultural Bank of China, and Bank of China became four major state-owned banks and were responsible for industrial and commercial credit businesses, urban construction financing, agricultural financing, and foreign currency exchange, respectively.

3 First-time macro-control and active internationalization (1993–2001)
  First-time macro-control occurred and financial supervision according to business type was established. In addition, foreign banks were permitted to operate.
4 Joining the World Trade Organization and establishing the China Banking Regulatory Commission (2002–2006)

The period of 2001 to 2006 during which China joined the World Trade Organization (WTO) was a transition phase for banking supervision in China. The open-door policies during this period were to implement liberalization measures based on WTO commitments fulfillment and the requirements of China’s reform and development.

In March 2003, the China Banking Regulatory Commission (CBRC) was founded and belonged to the State Council of the People’s Republic of China. The CBRC was responsible for integrating the People’s Bank of China with other banks, asset management companies, trust and investment companies, deposit-taking financial institutions, and the Central Financial Work Commission.

The responsibilities of the CBRC were to formulate related policies and regulations for banking supervision, to be responsible for market access and supervision operation, and to investigate and punish violations. The CBRC, the Securities and Futures Commission, and China Insurance Regulatory Commission supervised and regulated the banking, securities, and insurance sectors, respectively. Accordingly, a financial operation model for financial management and supervision based on business type was established in China [12–13].

5 Corporate orientation and financial globalization (after 2006)

In 2006, the Regulations on the Administration of Foreign-funded Banks of the People’s Republic of China were announced. Although foreign-funded banks were permitted to establish branches, subsidiary banks received more preferential treatment compared with bank branches pertaining to capital, review processes, business locations, currency type, business items, and target customers.

Although China currently performs supervision based on business type, various financial supervisory authorities have gradually established a joint supervision mechanism and exchanged supervisory information through a joint-meeting supervisory mechanism and a regular contact mechanism [12–13]. The main financial supervision laws involved are the Law of the People's Republic of China on the People's Bank of China, Law of the People’s Republic of China on Commercial Banks, Regulations on the Administration of Foreign-funded Banks of the People’s Republic of China, Law of the People’s Republic of China on Banking Regulation and Supervision, and Regulations on the Foreign Exchange System of the People’s Republic of China.

## Methods

The study was reviewed and approved by an institutional review board Zhongshan Institute, University of Electronic Science and Technology of China (ethics committee).

Previous studies on financial supervision criteria have a major shortcoming. Most studies have assumed that evaluation criteria are independent of one another and that no causal relationship exists among evaluation criteria. This assumption limited research on financial supervision criteria.

To establish evaluation criteria for banking supervision in China, this study integrated fuzzy theory and the DEMATEL and proposes a fuzzy-DEMATEL model. First, fuzzy theory was applied to examine banking supervision criteria and analyze fuzzy semantics. Second, the fuzzy-DEMATEL model was used to calculate the degree to which financial supervision criteria mutually influenced one another and their causal relationship. Finally, an evaluation criteria model for evaluating banking and financial supervision was established.

Following the discussed studies on how supervision agencies in various countries supervise banks and those on the criteria for evaluating banking supervision [14–17], we established an indicator system for assessing banking supervision.

This set of criteria included four dimensions (i.e., financial soundness, law compliance, consumer protection, and risk management) and 10 evaluation criteria, as shown in Table 1.

**Table 1:** Dimensions and criteria for banking supervision.

Evaluation dimensions and criteria	Dimension	Criteria
Evaluation criteria system for financial supervision	Financial soundness	Capital adequacy
		Asset quality
		Earnings
	Law compliance	Supervising the board of directors and management level
		Status of complying with major laws
	Consumer protection	Soundness of operation control
		Consumer complaints handling
	Risk management	Credit risk management
		Market risk management
		Operational risk management

### Decision Making Trial and Evaluation Laboratory

The DEMATEL was developed by the Battelle Memorial Institute at the Geneva Research Center. Gabus and Fontela [18–21] attempted to use the DEMATEL to solve the world’s intractable problems, such as problems related to race, hunger, environmental protection, and energy [22–26]. The purpose of the DEMATEL is to examine the relationship between attributes in a complex system and to apply matrix computation to derive the causal relationship between the attributes and degree to which the attributes influence one another. The DEMATEL can convert a complex system into a clearly structured causal relationship [27–30]. In addition, the DEMATEL can simplify the relationship between attributes in a complex system into two groups (causes and effects) and quantify the degree to which the attributes mutually influence one another and thus identify core problems for the complex system and improvement directions [31–33].

In recent years, the DEMATEL has been employed to solve various problems in various fields. Tzeng et al. [34] used the DEMATEL to assess e-learning effectiveness. Lin and Wu [35] adopted the fuzzy-DEMATEL to solve problems related to group decision making. Lee et al. [36] applied the DEMATEL to the technology acceptance model and examined application effectiveness. Lee and Huang [37] used the DEMATEL to analyze the causal relationship between service properties, to adjust the importance of service properties, and to solve core problems. Tsai et al. [38], based on the DEMATEL, investigated how manufacturing enterprises won orders and developed excellent competitive strategies. Lee et al. [27] employed a DEMATEL model to investigate the environmental performance of product suppliers.

### Fuzzy Theory

Zadeh [39] proposed fuzzy set theory and indicated that the thinking, reasoning, and understanding of people are fuzzy and subjective and cannot be easily quantified using precise ratios or figures. Therefore, Zadeh claimed that numerous conventional and precise quantitative methods cannot completely solve human-centered or other complex problems. To manage uncertainty and fuzziness in reality, fuzzy set theory is required. In other words, numerical values between 0 and 1 are used to indicate the degree of a research target’s fuzziness. The subjective judgments of people are converted into numerical values between 0 and 1 to overcome the shortcomings of two-valued logic, thereby yielding research results that are consistent with the characteristics of human thinking.

According to fuzzy logic, each numerical value between 0 and 1 is partially correct. In a crisp set, the value of an element is either 1 or 0. Therefore, fuzzy logic can manage fuzzy and indefinite mathematical judgment [40]. Triangular, trapezoidal, and Gaussian fuzzy numbers are commonly used fuzzy numbers. The function of fuzzy semantics is to convert semantic words into fuzzy numbers and then defuzzify the fuzzy numbers and obtain crisp values [41–42].

In this study, regarding the defuzzification procedure and solution derivation, the minimal and maximal fuzzy numbers were used to determine the right and left critical values. The total integral value was determined based on the weighted average of the membership function [43]. The implementation procedure included four steps, which are presented as follows [42–45]:

Step 1: Standardization
  $$r_{i}^{\max}=\max\;r_{j}^{i},\;l_{i}^{\min}=\min\;l_{j}^{i},\;\Delta_{\min}^{\max}=\min l_{ij}$$
  Calculate all solutions: *a*_*j*_, *j = 1*, *…*, *J*
  $$x_{lj}=(l_{ij}-l_{i}^{\min})/\Delta_{\min}^{\max}\;x_{mj}=(m_{ij}-l_{i}^{\min})/\Delta_{\min}^{\max}\;x_{rj}=(r_{ij}-l_{i}^{\min})/\Delta_{\min}^{\max}$$ (1)
Step 2: Calculate the left and right normalized critical values, *j = 1*, *…*, *J*.
  $$X_{j}^{ls}=x_{mj}/1+x_{mj}-x_{lj}\;X_{j}^{rs}=x_{rj}/1+x_{rj}-x_{mj}$$ (2)
Step 3: Calculate all normalized crisp values, *j = 1*, *…*, *J*.
  $$x_{j}^{crisp}=\left[x_{j}^{ls}(1-x_{j}^{ls})+x_{j}^{rs}x_{j}^{rs}\right]/\left[1-x_{j}^{ls}+x_{j}^{rs}\right]$$ (3)
Step 4: Calculate crisp values, *j = 1*, *…*, *J*.
  $$f_{ij}=l_{i}^{\min}+x_{j}^{crisp}\Delta_{\min}^{\max}$$ (4)

### Fuzzy DEMATEL

The fuzzy-DEMATEL model is an integration of fuzzy semantics and the DEMATEL method. In other words, by applying the DEMATEL method to a fuzzy situation, a causal relationship between variables can be examined in the fuzzy situation and how variables mutually influence one another can be clarified. The computation procedure for the fuzzy-DEMATEL model is elaborated as follows:

Step 1: Develop evaluation criteria and design a fuzzy semantic scale.

Instead of a conventional scale, a fuzzy semantic scale was used to solve fuzzy problems related to human thinking. In this study, according to Li and Tzeng (2009), triangular fuzzy numbers were used to determine the degree to which variables mutually influenced one another (Table 2); (0.0, 0.0, 0.0) represents no influence, (0, 0.25, 0.5) represents very low influence, (0.25, 0.5, 0.75) represents low influence, (0.5, 0.75, 1.0) represents high influence, and (0.75, 1.0, 1.0) represents very high influence.

**Table 2:** Fuzzy semantics.

Degree of influence	Score	Triangular fuzzy number
Very high influence (VH)	4	(0.75, 1.0, 1.0)
High influence (H)	3	(0.5, 0.75 1.0)
Low influence (L)	2	(0.25, 0.5, 0.75)
Very low influence (VL)	1	(0.0, 0.25, 0.5)
No influence (No)	0	(0.0, 0.0, 0.0)

Step 2: Summarize the evaluation results obtained by experts.

To evaluate the relationship between various criteria *C* = {*C*_*i*_|*i* = 1,2,…,*n*}, *p* experts used the fuzzy sematic scale to measure the degree to which two criteria mutually influenced each other. Therefore, *p* fuzzy matrices (${\tilde{Z}}^{\left(1\right)}$, ${\tilde{Z}}^{\left(2\right)}$, …, ${\tilde{Z}}^{\left(p\right)}$) were obtained. The fuzzy matrix ${\tilde{Z}}^{\left(K\right)}$ is expressed as follows:

$${\tilde{Z}}^{\left(K\right)}=\left[\begin{array}{llll} 0 & {\tilde{z}}_{12}^{\left(k\right)} & \cdots & {\tilde{z}}_{1n}^{\left(k\right)}\\ {\tilde{z}}_{21}^{\left(k\right)} & 0 & \cdots & {\tilde{z}}_{2n}^{\left(k\right)}\\ \vdots & \vdots & \ddots & \vdots \\ {\tilde{z}}_{n1}^{\left(k\right)} & {\tilde{z}}_{n2}^{\left(k\right)} & \cdots & 0 \end{array}\right]\;;\;k=1,2,\cdots ,p,$$ (5)

where $z_{ij}^{\left(k\right)}=\left(l_{ij}^{\left(k\right)},m_{ij}^{\left(K\right)},u_{ij}^{\left(k\right)}\right)$, is a triangular fuzzy number (0,0,0), and ${\tilde{Z}}^{\left(K\right)}$ denotes the fuzzy matrix for the initial direct relationship derived from the judgment of the *k*^th^ expert.

Step 3: Establish a standardized fuzzy matrix for a direct relationship.

Let ${\tilde{a}}_{i}^{\left(k\right)}$ be a triangular fuzzy function:

$${\tilde{a}}_{i}^{\left(k\right)}=\sum_{j=1}^{n}{\tilde{z}}_{ij}^{\left(k\right)}=\left(\sum_{j=1}^{n}l_{ij}^{\left(k\right)},\sum_{j=1}^{n}m_{ij}^{\left(k\right)},\sum_{j=1}^{n}u_{ij}^{\left(k\right)}\right)\;And$$

$$r^{\left(k\right)}=\underset{1\leq i\leq n}{\max}\left(\sum_{j=1}^{n}u_{ij}^{\left(k\right)}\right)$$

By linear conversion, the fuzzy matrix for a direct relationship can be standardized and expressed as follows:

$${\tilde{X}}^{\left(k\right)}=\left[\begin{array}{llll} {\tilde{x}}_{11}^{\left(k\right)} & {\tilde{x}}_{21}^{\left(k\right)} & \cdots & {\tilde{x}}_{1n}^{\left(k\right)}\\ {\tilde{x}}_{21}^{\left(k\right)} & x_{22}^{\left(k\right)} & \cdots & {\tilde{x}}_{2n}^{\left(k\right)}\\ \vdots & \vdots & \ddots & \vdots \\ {\tilde{x}}_{n1}^{\left(k\right)} & {\tilde{x}}_{22}^{\left(k\right)} & \cdots & {\tilde{x}}_{nn}^{\left(k\right)} \end{array}\right]\;;\;k=1,2,\cdots ,p,$$ (6)

where x˜ij(K)=z˜ij(k)r(k)=(lij(k)r(k),mij(k)r(k),uij(k)r(k)).

According to the DEMATEL, the assumption $\sum_{j=1}^{n}u_{ij}^{\left(k\right)}$<*r*^(*k*)^. must be met. By applying matrix computation, the average matrix ***X*** can be obtained.

Step 4: Construct the fuzzy matrix for a complete relationship.

To construct the fuzzy matrix for a complete relationship ***T***, the condition $\underset{w\to \infty }{\lim}{\tilde{X}}^{w}=0$ must be met. The symbol ${\tilde{X}}^{w}$ denotes a triangular fuzzy matrix and is expressed as follows:

$${\tilde{X}}^{\left(w\right)}=\left[\begin{array}{llll} {\tilde{x}}_{11}^{\left(w\right)} & {\tilde{x}}_{21}^{\left(w\right)} & \cdots & {\tilde{x}}_{1n}^{\left(w\right)}\\ {\tilde{x}}_{21}^{\left(w\right)} & x_{22}^{\left(w\right)} & \cdots & {\tilde{x}}_{2n}^{\left(w\right)}\\ \vdots & \vdots & \ddots & \vdots \\ {\tilde{x}}_{n1}^{\left(w\right)} & {\tilde{x}}_{22}^{\left(w\right)} & \cdots & {\tilde{x}}_{nn}^{\left(w\right)} \end{array}\right],\;{\tilde{x}}_{ij}^{\left(w\right)}=(l_{ij}^{\left(w\right)},m_{ij}^{\left(w\right)},u_{ij}^{\left(w\right)})$$

According to the aforementioned Theorem 3.1, the fuzzy matrix can be decomposed as follows:

$$\left[l_{ij}^{\left(w\right)}\right]=\left[\begin{array}{llll} l_{11}^{\left(w\right)} & l_{12}^{\left(w\right)} & \cdots & l_{1n}^{\left(w\right)}\\ l_{21}^{\left(w\right)} & l_{22}^{\left(w\right)} & \cdots & l_{2n}^{\left(w\right)}\\ \vdots & \vdots & \ddots & \vdots \\ l_{n1}^{\left(w\right)} & l_{22}^{\left(w\right)} & \cdots & l_{nn}^{\left(w\right)} \end{array}\right]\;\left[m_{ij}^{\left(w\right)}\right]=\left[\begin{array}{llll} m_{11}^{\left(w\right)} & m_{12}^{\left(w\right)} & \cdots & m_{1n}^{\left(w\right)}\\ m_{21}^{\left(w\right)} & m_{22}^{\left(w\right)} & \cdots & m_{2n}^{\left(w\right)}\\ \vdots & \vdots & \ddots & \vdots \\ m_{n1}^{\left(w\right)} & m_{22}^{\left(w\right)} & \cdots & m_{nn}^{\left(w\right)} \end{array}\right]\;\left[u_{ij}^{\left(w\right)}\right]=\left[\begin{array}{llll} u_{11}^{\left(w\right)} & u_{12}^{\left(w\right)} & \cdots & u_{1n}^{\left(w\right)}\\ u_{21}^{\left(w\right)} & u_{22}^{\left(w\right)} & \cdots & u_{2n}^{\left(w\right)}\\ \vdots & \vdots & \ddots & \vdots \\ u_{n1}^{\left(w\right)} & u_{22}^{\left(w\right)} & \cdots & u_{nn}^{\left(w\right)} \end{array}\right]$$ (7)

The three matrices are expressed as follows:

$\left[l_{ij}^{\left(w\right)}\right]=X_{l}^{w},\left[m_{ij}^{\left(w\right)}\right]=X_{m}^{w},\left[u_{ij}^{\left(w\right)}\right]=X_{u}^{w}$ Let $\underset{w\to \infty }{\lim}X_{}^{w}=O$ and $\underset{w\to \infty }{\lim}\left(I+X+X^{2}+\cdots +X^{k}\right)={\left(I-X\right)}^{-1}$, where ***O*** is a zero matrix and ***I*** is an identity matrix.

$$\tilde{T}=\underset{w\to \infty }{\lim}\left(\tilde{X}+{\tilde{X}}^{2}+\cdots +{\tilde{X}}^{k}\right)=\tilde{X}{\left(I-\tilde{X}\right)}^{-1}$$ (8)

The normalized fuzzy matrix for a direct relationship is a convergent matrix. A fuzzy matrix *T* for a direct, indirect, or complete relationship can be expressed as follows:

$\tilde{T}=\underset{w\to \infty }{\lim}\left(\tilde{X}+{\tilde{X}}^{2}+\cdots +{\tilde{X}}^{k}\right)=\tilde{X}{\left(I-\tilde{X}\right)}^{-1}$ Let

$$\tilde{T}=\left[\begin{array}{llll} {\tilde{t}}_{11} & {\tilde{t}}_{12} & \cdots & {\tilde{t}}_{1n}\\ {\tilde{t}}_{21} & t_{22} & \cdots & {\tilde{t}}_{2n}\\ \vdots & \vdots & \ddots & \vdots \\ t_{n1} & t_{n2} & \cdots & {\tilde{t}}_{nn} \end{array}\right]$$

where ${\tilde{t}}_{ij}=\left(l_{ij}^{''},m_{ij}^{''},u_{ij}^{''}\right)$. Therefore,

$$\begin{array}{l} \;Matrix\left[l_{ij}^{''}\right]=X_{l}\times {\left(I-X_{l}\right)}^{-1}\\ Matrix\left[m_{ij}^{''}\right]=X_{m}\times {\left(I-X_{m}\right)}^{-1}\\ Matrix\left[u_{ij}^{''}\right]=X_{u}\times {\left(I-X_{u}\right)}^{-1} \end{array}$$ (9)

Step 5: Draw a causality diagram.

Integrate the three matrices into a fuzzy matrix for a complete relationship and use the converting fuzzy data into crisp scores technique for defuzzification. Convert fuzzy semantic values into crisp values and draw a causality diagram to determine the causal relationship between criteria and the degree to which criteria mutually influence one another.

## Results and Discussion

### Questionnaire survey

The questionnaire regarding the evaluation criteria system for financial supervision comprised four dimensions (financial soundness, law compliance, consumer protection, and risk management) and 10 evaluation criteria (a1: capital adequacy, a2: asset quality, a3: earnings, b1: supervising the board of directors and management level, b2: status of complying with major laws, c1: soundness of operation control, c2: consumer complaints handling, d1: credit risk management, d2: market risk management, d3: operational risk management).

DEMATEL questionnaires were distributed between August 1 and August 10, 2014. For each question, a 5-point scale (4–0) is used to indicate level of influence of each criterion. The scale anchors are in the descending order of very high influence (VH), high influence (H), low influence (L), very low influence (VL), and no influence (No). In this study, a group of Chinese experts provided their viewpoints regarding evaluation criteria for banking supervision in China. The questionnaires were distributed to 12 experts (5 professors of finance, 5 top managers in the banking sector, and 2 government officials in regulatory units). The author visited each expert in person, explained the content of the questionnaire, and requested each expert to complete the questionnaire. Overall, 12 questionnaires were distributed and returned. The valid return rate was 100%.

### Results

In this study, 12 experts were invited to provide expert opinions on supervision criteria. Based on the questionnaire results, the causal relationship between criteria and the degree to which criteria mutually influenced one another were calculated. Table 3 shows the average scores of the experts.

**Table 3:** Fuzzy relationship between criteria.

Criteria	a1	a2	a3	b1	b2	c1	c2	d1	d2	d3
a1	0	L	L	L	L	H	H	L	H	L
a2	VL	0	VL	L	0	H	L	VL	VL	VL
a3	VL	VL	0	O	O	VL	VL	VL	VL	VL
b1	L	L	VL	0	H	H	VH	L	L	L
b2	0	0	VL	VL	0	VL	VL	0	0	VL
c1	0	0	VL	0	VL	0	VL	VL	0	0
c2	0	0	0	0	VL	VL	0	0	0	VL
d1	0	L	L	L	L	H	H	0	L	L
d2	0	0	0	0	L	L	L	0	0	VL
d3	0	0	0	0	L	L	L	0	VL	0

This study used Matlab software to calculate.The fuzzy scale in Table 3 was converted into fuzzy values, as shown in Table 4. In this study, five semantic phrases were used to express the degree of influence (very high influence (VH), high influence (H), low influence (L), very low influence (VL), and no influence (No)). By using Eq (1), fuzzy semantic values VH, H, L, VL, and No were converted into (0.75, 1.0, 10.0), (0.5, 0.75, 1.0), (0.25, 0.5, 0.75), (0, 0.25, 0.5), and (0, 0, 0), respectively. Thus, a fuzzy matrix for a direct relationship was established, as shown in Table 5.

**Table 4:** Fuzzy scale converted into fuzzy values.

Criteria	a1	a2	a3	b1	b2	c1	c2	d1	d2	d3
a1	(0.0, 0.0, 0.0)	(0.25, 0.5, 0.75)	(0.25, 0.5, 0.75)	(0.25, 0.5, 0.75)	(0.25, 0.5, 0.75)	(0.5, 0.75 1.0)	(0.5, 0.75 1.0)	(0.25, 0.5, 0.75)	(0.5, 0.75 1.0)	(0.25, 0.5, 0.75)
a2	(0.0, 0.25, 0.5)	(0.0, 0.0, 0.0)	(0.0, 0.25, 0.5)	(0.25, 0.5, 0.75)	(0.0, 0.0, 0.0)	(0.5, 0.75 1.0)	(0.25, 0.5, 0.75)	(0.0, 0.25, 0.5)	(0.0, 0.25, 0.5)	(0.0, 0.25, 0.5)
a3	(0.0, 0.25, 0.5)	(0.0, 0.25, 0.5)	(0.0, 0.0, 0.0)	(0.0, 0.0, 0.0)	(0.0, 0.0, 0.0)	(0.0, 0.25, 0.5)	(0.0, 0.25, 0.5)	(0.0, 0.25, 0.5)	(0.0, 0.25, 0.5)	(0.0, 0.25, 0.5)
b1	(0.25, 0.5, 0.75)	(0.25, 0.5, 0.75)	(0.0, 0.25, 0.5)	(0.0, 0.0, 0.0)	(0.5, 0.75 1.0)	(0.5, 0.75 1.0)	(0.75, 1.0, 1.0)	(0.25, 0.5, 0.75)	(0.25, 0.5, 0.75)	(0.25, 0.5, 0.75)
b2	(0.0, 0.0, 0.0)	(0.0, 0.0, 0.0)	(0.0, 0.25, 0.5)	(0.0, 0.25, 0.5)	(0.0, 0.0, 0.0)	(0.0, 0.25, 0.5)	(0.0, 0.25, 0.5)	(0.0, 0.0, 0.0)	(0.0, 0.0, 0.0)	(0.0, 0.25, 0.5)
c1	(0.0, 0.0, 0.0)	(0.0, 0.0, 0.0)	(0.0, 0.25, 0.5)	(0.0, 0.0, 0.0)	(0.0, 0.25, 0.5)	(0.0, 0.0, 0.0)	(0.0, 0.25, 0.5)	(0.0, 0.25, 0.5)	(0.0, 0.0, 0.0)	(0.0, 0.0, 0.0)
c2	(0.0, 0.0, 0.0)	(0.0, 0.0, 0.0)	(0.0, 0.0, 0.0)	(0.0, 0.0, 0.0)	(0.0, 0.25, 0.5)	(0.0, 0.25, 0.5)	(0.0, 0.0, 0.0)	(0.0, 0.0, 0.0)	(0.0, 0.0, 0.0)	(0.0, 0.25, 0.5)
d1	(0.0, 0.0, 0.0)	(0.25, 0.5, 0.75)	(0.25, 0.5, 0.75)	(0.25, 0.5, 0.75)	(0.25, 0.5, 0.75)	(0.5, 0.75 1.0)	(0.5, 0.75 1.0)	(0.0, 0.0, 0.0)	(0.25, 0.5, 0.75)	(0.25, 0.5, 0.75)
d2	(0.0, 0.0, 0.0)	(0.0, 0.0, 0.0)	(0.0, 0.0, 0.0)	(0.0, 0.0, 0.0)	(0.25, 0.5, 0.75)	(0.25, 0.5, 0.75)	(0.25, 0.5, 0.75)	(0.0, 0.0, 0.0)	(0.0, 0.0, 0.0)	(0.0, 0.25, 0.5)
d3	(0.0, 0.0, 0.0)	(0.0, 0.0, 0.0)	(0.0, 0.0, 0.0)	(0.0, 0.0, 0.0)	(0.25, 0.5, 0.75)	(0.25, 0.5, 0.75)	(0.25, 0.5, 0.75)	(0.0, 0.0, 0.0)	(0.0, 0.25, 0.5)	(0.0, 0.0, 0.0)

**Table 5:** Fuzzy matrix for a direct relationship.

Criteria	a1			a2			a3			b1			b2			c1			c2			d1			d2			d3		
	x	y	z	x	y	z	x	y	z	x	y	z	x	y	z	x	y	z	x	y	z	x	y	z	x	y	z	x	y	z
a1	0	0	0	0.25	0.5	0.75	0.25	0.5	0.75	0.25	0.5	0.75	0.25	0.5	0.75	0.5	0.75	1.0	0.5	0.75	1.0	0.25	0.5	0.75	0.5	0.75	1.0	0.25	0.5	0.75
a2	0	0.25	0.5	0	0	0	0	0.25	0.5	0.25	0.5	0.75	0	0	0	0.5	0.75	1.0	0.25	0.5	0.75	0	0.25	0.5	0	0.25	0.5	0	0.25	0.5
a3	0	0.25	0.5	0	0.25	0.5	0	0	0	0	0	0	0	0	0	0	0.25	0.5	0	0.25	0.5	0	0.25	0.5	0	0.25	0.5	0	0.25	0.5
b1	0.25	0.5	0.75	0.25	0.5	0.75	0	0.25	0.5	0	0	0	0.5	0.75	1.0	0.5	0.75	1.0	0.75	1.0	1.0	0.25	0.5	0.75	0.25	0.5	0.75	0.25	0.5	0.75
b2	0	0	0	0	0	0	0	0.25	0.5	0	0.25	0.5	0	0	0	0	0.25	0.5	0	0.25	0.5	0	0	0	0	0	0	0	0.25	0.5
c1	0	0	0	0	0	0	0	0.25	0.5	0	0	0	0	0.25	0.5	0	0	0	0	0.25	0.5	0	0.25	0.5	0	0	0	0	0	0
c2	0	0	0	0	0	0	0	0	0	0	0	0	0	0.25	0.5	0	0.25	0.5	0	0	0	0	0	0	0	0	0	0	0.25	0.5
d1	0	0	0	0.25	0.5	0.75	0.25	0.5	0.75	0.25	0.5	0.75	0.25	0.5	0.75	0.5	0.75	1.0	0.5	0.75	1.0	0	0	0	0.25	0.5	0.75	0.25	0.5	0.75
d2	0	0	0	0	0	0	0	0	0	0	0	0	0.25	0.5	0.75	0.25	0.5	0.75	0.25	0.5	0.75	0	0	0	0	0	0	0	0.25	0.5
d3	0	0	0	0	0	0	0	0	0	0	0	0	0.25	0.5	0.75	0.25	0.5	0.75	0.25	0.5	0.75	0	0	0	0	0.25	0.5	0	0	0

Subsequently, Eqs (5)–(8) were used to construct three fuzzy matrices for a direct relationship (*X*_*l*_, *X*_*m*_, *X*_*n*_) and then the matrices were normalized. The fuzzy matrices for a direct relationship (*X*_*l*_, *X*_*m*_, *X*_*n*_) were derived based on the column vector *u* (*l* ≤ *m* ≤ *u*) and the maximal value in *u*.

By using Eq (9), the matrices *X*_*l*_, *X*_*m*_ and *X*_*n*_ for a complete relationship were integrated and (D + R) and (D—R) were calculated, as shown in Table 6.

**Table 6:** Prominence and relation for fuzzy-DEMATEL.

Criteria	D			R			D + R			D—R		
	x	y	z	x	y	z	x	y	z	x	y	z
a1	0.44	0.97	2.50	0.04	0.18	1.13	0.48	1.15	3.63	-0.68	0.80	2.47
a2	0.15	0.58	1.82	0.11	0.30	1.34	0.26	0.88	3.16	-1.19	0.28	1.71
b1	0.00	0.34	1.41	0.07	0.37	1.46	0.07	0.71	2.87	-1.46	-0.03	1.34
b2	0.44	0.96	2.43	0.11	0.31	1.35	0.55	1.27	3.78	-0.91	0.66	2.32
b3	0.00	0.23	1.22	0.22	0.62	1.89	0.22	0.85	3.10	-1.89	-0.39	1.00
c1	0.00	0.18	1.14	0.36	0.87	2.33	0.36	1.05	3.46	-2.33	-0.68	0.77
c2	0.00	0.12	1.03	0.36	0.87	2.25	0.36	0.99	3.29	-2.25	-0.74	0.67
c3	0.36	0.81	2.22	0.07	0.32	1.37	0.43	1.13	3.59	-1.01	0.49	2.15
d1	0.10	0.28	1.31	0.14	0.43	1.57	0.24	0.71	2.88	-1.47	-0.15	1.16
d2	0.10	0.28	1.31	0.11	0.51	1.70	0.21	0.78	3.00	-1.60	-0.23	1.20

Finally, Eqs (7)–(9) were used for defuzzification, and fuzzy semantic values were converted into crisp values. Prominence ((D + R)_def_ and relation ((D - R)_def_ were then calculated, as shown in Table 7.

**Table 7:** Prominence and relation following defuzzification.

Criteria	(Di + Ri)	(Di—Ri)
a1	1.75	0.86
a2	1.43	0.27
b1	1.22	-0.05
b2	1.87	0.69
b3	1.39	-0.43
c1	1.63	-0.74
c2	1.55	-0.78
c3	1.72	0.54
d1	1.28	-0.15
d2	1.33	-0.21
Average	1.52	0.00

**Fig 1:**
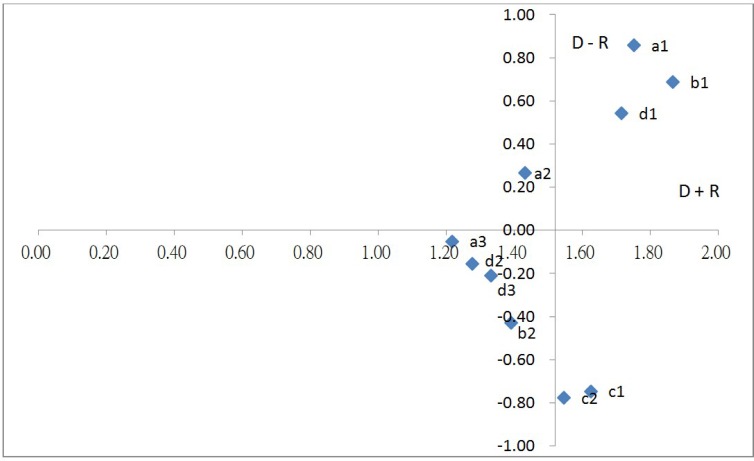
Relationship between criteria.

By using (D + R)_def_ as the horizontal axis and (D-R)_def_ as the vertical axis, 10 criteria were plotted, as shown in Fig 1.

### Discussion

Based on relation (D - R) and prominence (D + R), a detailed discussion of the 10 criteria and their causal relationship and mutual influence, as shown in Table 6 and Fig 1, is presented.

1. High relation and high prominence: Capital adequacy (a1), supervising the board of directors and management level (b1), and credit risk management (d1) appeared in this quadrant. These three attributes were in the category of causes and were the core items that influenced other attributes. The three attributes were key factors for solving problems.
2. High relation and low prominence: Only asset quality (a2) appeared in this quadrant. This attribute influenced other attributes to a low degree.
3. Low relation and high prominence: Soundness of operation control (c1) and consumer complaints handling (c2) appeared in this quadrant. These two attributes were in the category of effects and were influenced by other attributes. The two attributes could not be directly improved.
4. Low relation and low prominence: Earnings (a3), status of complying with major laws (b2), market risk management, and operational risk management (d3) appeared in this quadrant. These four attributes were influenced by other attributes to an exceedingly low degree, suggesting that these attributes were relatively independent.

Regarding the degree of influence and causal relationship, we determined that capital adequacy (a1), supervising the board of directors and management level (b1), and credit risk management (d1) were three crucial and core attributes that influenced other attributes and were the key factors for solving problems. According to practical experiences in financial supervision, capital adequacy (a1) is the basis for the equity capital and liquidity of a bank and is the most crucial factor influencing a bank’s constitution. If a bank has high equity capital, then the bank would have a low risk of closure. In addition, supervising the board of directors and management level (b1) is crucial for banking supervision. The fraud processes of a bank typically involve the board of directors and management level for the bank, and therefore, all competent authorities for financial institutions rigorously supervise the board of directors and management level for banks. Moreover, credit risk management (d1) is a crucial criterion for financial supervision. A bank’s credit risk should remain within a safe range to avoid a credit crisis for the bank. By enhancing the performance of the aforementioned three criteria, the performance of other criteria for banking supervision can be improved.

## Conclusion

Financial supervision means that monetary authorities have the power to supervise and manage financial institutions according to laws. Monetary authorities have this power because of the requirements of improving financial services, protecting the rights of depositors, adapting to industrial development, ensuring financial fair trade, and maintaining stable financial order. The passive function of financial supervision is to improve the operation of financial services and to maintain a stable financial order. The positive function is to protect the rights of depositors and ensure fair trade for financial systems according to financial environments.

Previous studies on financial supervision criteria have a major shortcoming. Most studies have assumed that evaluation criteria are independent of one another and that no causal relationship exists between evaluation criteria. This assumption limited research on financial supervision criteria. To establish evaluation criteria for banking supervision in China, this study integrated fuzzy theory and the DEMATEL and proposes a fuzzy-DEMATEL model. First, fuzzy theory was applied to examine banking supervision criteria and analyze fuzzy semantics. Second, the fuzzy-DEMATEL model was used to calculate the degree to which financial supervision criteria mutually influenced one another and their causal relationship. Finally, an evaluation criteria model for evaluating banking and financial supervision was established.

This study determined that capital adequacy (a1), supervising the board of directors and management level (b1), and credit risk management (d1) were three crucial and core attributes that influenced other attributes and were the key factors for solving problems. By enhancing the performance of the three financial supervisory criteria, the performance of other criteria for banking supervision can be improved.

The fuzzy-DEMATEL model can be used to determine the causal relationships among criteria and the influence of the criteria; to structure complex problems; and to identify, clarify, and improve problems. However, the mathematical model and computation procedures are complex; accordingly, users must pay special attention. In addition, we suggest that future studies adopt other evaluation methods to perform integrative analyses and compare the strengths and weaknesses of various methods.

## Supporting Information

S1 File Questionnaire–English.docs. (DOCX)

S2 File Questionnaire–Chinese.docs. (DOCX)
