# Supplementary material for: Using a Mixed Model to Explore Evaluation Criteria for Bank Supervision: A Banking Supervision Law Perspective
Source: PLoS One. 2016 Dec 19;11(12):e0167710. doi: 10.1371/journal.pone.0167710 (PMC5167262; doi:10.1371/journal.pone.0167710)
Supplement: S1 File — (DOCX) [file pone.0167710.s001.docx]

**Exploring Evaluation Criteria for Bank Supervision**

**Questionnaire Survey**

一、Introduction

Dear Expert:

We are writing to you because of your expertise in your field. In our study, we are exploring the evaluation criteria used in banking supervision. Because we have adopted the expert method to understand the perspectives of experts in the field, we invite you to complete this questionnaire. Your responses will be used only for academic research purposes.

Zhongshan Institute, University of Electronic Science and Technology of China

Prof. Sang-Bing Tsai

1. Instructions

(1) This questionnaire was designed to collect opinions from experts regarding an issue. Your responses will be subjected to an analysis, the results of which will serve as the basis of our research.

(2) For each question, a 5-point scale is used to indicate level of influence of each criterion. The scale anchors are in the descending order of *very high influence* (VH), *high influence* (H), *low influence* (L), *very low influence* (VL), and *no influence* (No). Please complete each question by answering “VH,” “H,” “L,” “VL,” or “No” to indicate the level of influence that each listed criterion has on the other 10 criteria.

三、Questionnaire content

1. Please indicate the level of influence that capital adequacy has on the other 10 criteria:

| Criteria | Capital adequacy | Asset quality | Earnings | Supervising the board of directors and management level | Status of complying with major laws | Soundness of operation control | Consumer complaints handling | Credit risk management | Market risk management | Operational risk management |
| --- | --- | --- | --- | --- | --- | --- | --- | --- | --- | --- |
| Capital adequacy | No |  |  |  |  |  |  |  |  |  |

2. Please indicate the level of influence that asset quality has on the other 10 criteria:

| Criteria | Capital adequacy | Asset quality | Earnings | Supervising the board of directors and management level | Status of complying with major laws | Soundness of operation control | Consumer complaints handling | Credit risk management | Market risk management | Operational risk management |
| --- | --- | --- | --- | --- | --- | --- | --- | --- | --- | --- |
| Asset quality |  | No |  |  |  |  |  |  |  |  |

3. Please indicate the level of influence that earnings has on the other 10 criteria:

| Criteria | Capital adequacy | Asset quality | Earnings | Supervising the board of directors and management level | Status of complying with major laws | Soundness of operation control | Consumer complaints handling | Credit risk management | Market risk management | Operational risk management |
| --- | --- | --- | --- | --- | --- | --- | --- | --- | --- | --- |
| Earnings |  |  | No |  |  |  |  |  |  |  |

4. Please indicate the level of influence that supervising the board of directors and management level has on the other 10 criteria:

| Criteria | Capital adequacy | Asset quality | Earnings | Supervising the board of directors and management level | Status of complying with major laws | Soundness of operation control | Consumer complaints handling | Credit risk management | Market risk management | Operational risk management |
| --- | --- | --- | --- | --- | --- | --- | --- | --- | --- | --- |
| Supervising the board of directors and management level |  |  |  | No |  |  |  |  |  |  |

5. Please indicate the level of influence that the status of complying with major laws has on the other 10 criteria:

| Criteria | Capital adequacy | Asset quality | Earnings | Supervising the board of directors and management level | Status of complying with major laws | Soundness of operation control | Consumer complaints handling | Credit risk management | Market risk management | Operational risk management |
| --- | --- | --- | --- | --- | --- | --- | --- | --- | --- | --- |
| Status of complying with major laws |  |  |  |  | No |  |  |  |  |  |

6. Please indicate the level of influence that soundness of operation control has on the other 10 criteria:

| Criteria | Capital adequacy | Asset quality | Earnings | Supervising the board of directors and management level | Status of complying with major laws | Soundness of operation control | Consumer complaints handling | Credit risk management | Market risk management | Operational risk management |
| --- | --- | --- | --- | --- | --- | --- | --- | --- | --- | --- |
| Soundness of operation control |  |  |  |  |  | No |  |  |  |  |

7. Please indicate the level of influence that consumer complaints handling on other 10 criteria:

| Criteria | Capital adequacy | Asset quality | Earnings | Supervising the board of directors and management level | Status of complying with major laws | Soundness of operation control | Consumer complaints handling | Credit risk management | Market risk management | Operational risk management |
| --- | --- | --- | --- | --- | --- | --- | --- | --- | --- | --- |
| Consumer complaints handling |  |  |  |  |  |  | No |  |  |  |

8. Please indicate the level of influence that credit risk management has on the other 10 criteria:

| Criteria | Capital adequacy | Asset quality | Earnings | Supervising the board of directors and management level | Status of complying with major laws | Soundness of operation control | Consumer complaints handling | Credit risk management | Market risk management | Operational risk management |
| --- | --- | --- | --- | --- | --- | --- | --- | --- | --- | --- |
| Credit risk management |  |  |  |  |  |  |  | No |  |  |

9. Please indicate the level of influence that market risk management has on the other 10 criteria:

| Criteria | Capital adequacy | Asset quality | Earnings | Supervising the board of directors and management level | Status of complying with major laws | Soundness of operation control | Consumer complaints handling | Credit risk management | Market risk management | Operational risk management |
| --- | --- | --- | --- | --- | --- | --- | --- | --- | --- | --- |
| Market risk management |  |  |  |  |  |  |  |  | No |  |

10. Please indicate the level of influence that operational risk management has on the other 10 criteria:

| Criteria | Capital adequacy | Asset quality | Earnings | Supervising the board of directors and management level | Status of complying with major laws | Soundness of operation control | Consumer complaints handling | Credit risk management | Market risk management | Operational risk management |
| --- | --- | --- | --- | --- | --- | --- | --- | --- | --- | --- |
| Operational risk management |  |  |  |  |  |  |  |  |  | No |

Thank you for your cooperation.
