# Supplementary material for: Using a Mixed Model to Explore Evaluation Criteria for Bank Supervision: A Banking Supervision Law Perspective
Source: PLoS One. 2016 Dec 19;11(12):e0167710. doi: 10.1371/journal.pone.0167710 (PMC5167262; doi:10.1371/journal.pone.0167710)
Supplement: S2 File — (DOCX) [file pone.0167710.s002.docx]

**Exploring Evaluation Criteria for Bank Supervision**

**問卷調查表**

一、研究說明

敬愛的專家，您好：

素仰尊座學有專精，於本領域享有盛譽，本研究主要探討中國銀行監理評估指標，邀請您填答問卷。本研究採用專家意見研究調查方式法擬了解相關訊息。希望藉由您的寶貴經驗，僅提供後學學術研究之用，請您惠予作答。

Zhongshan Institute, University of Electronic Science and Technology of China

Prof. Sang-Bing Tsai

1. 填答說明

(1) 本研究係透過一群專家對同一主題看法提出個人意見，經研究彙整分析，作為研究之重要依據。

(2) 本研究評分方式為5分制，以專家個人意見表達影響程度，Very high influence (VH)代表影響程度最大，No Influence (No)代表影響程度最小。中間分數則按重要程度，分別以High Influence (H)、 Low Influence (L)、 Very Iow Influence (VL)加以評分。請依您的意見評判其適切性，請將您寶貴的意見在矩陣□中填寫VH、H、L、VL、No等意見。

三、問卷內容

1. Capital adequacy對其他Criteria的影響程度

| Criteria | Capital adequacy | Asset quality | Earnings | Supervising the board of directors and management level | Status of complying with major laws | Soundness of operation control | Consumer complaints handling | Credit risk management | Market risk management | Operational risk management |
| --- | --- | --- | --- | --- | --- | --- | --- | --- | --- | --- |
| Capital adequacy | No |  |  |  |  |  |  |  |  |  |

2. Asset quality對其他Criteria的影響程度

| Criteria | Capital adequacy | Asset quality | Earnings | Supervising the board of directors and management level | Status of complying with major laws | Soundness of operation control | Consumer complaints handling | Credit risk management | Market risk management | Operational risk management |
| --- | --- | --- | --- | --- | --- | --- | --- | --- | --- | --- |
| Asset quality |  | No |  |  |  |  |  |  |  |  |

3. Earnings對其他Criteria的影響程度

| Criteria | Capital adequacy | Asset quality | Earnings | Supervising the board of directors and management level | Status of complying with major laws | Soundness of operation control | Consumer complaints handling | Credit risk management | Market risk management | Operational risk management |
| --- | --- | --- | --- | --- | --- | --- | --- | --- | --- | --- |
| Earnings |  |  | No |  |  |  |  |  |  |  |

4. Supervising the board of directors and management level對其他Criteria的影響程度

| Criteria | Capital adequacy | Asset quality | Earnings | Supervising the board of directors and management level | Status of complying with major laws | Soundness of operation control | Consumer complaints handling | Credit risk management | Market risk management | Operational risk management |
| --- | --- | --- | --- | --- | --- | --- | --- | --- | --- | --- |
| Supervising the board of directors and management level |  |  |  | No |  |  |  |  |  |  |

5. Status of complying with major laws對其他Criteria的影響程度

| Criteria | Capital adequacy | Asset quality | Earnings | Supervising the board of directors and management level | Status of complying with major laws | Soundness of operation control | Consumer complaints handling | Credit risk management | Market risk management | Operational risk management |
| --- | --- | --- | --- | --- | --- | --- | --- | --- | --- | --- |
| Status of complying with major laws |  |  |  |  | No |  |  |  |  |  |

6. Soundness of operation control對其他Criteria的影響程度

| Criteria | Capital adequacy | Asset quality | Earnings | Supervising the board of directors and management level | Status of complying with major laws | Soundness of operation control | Consumer complaints handling | Credit risk management | Market risk management | Operational risk management |
| --- | --- | --- | --- | --- | --- | --- | --- | --- | --- | --- |
| Soundness of operation control |  |  |  |  |  | No |  |  |  |  |

7. Consumer complaints handling對其他Criteria的影響程度

| Criteria | Capital adequacy | Asset quality | Earnings | Supervising the board of directors and management level | Status of complying with major laws | Soundness of operation control | Consumer complaints handling | Credit risk management | Market risk management | Operational risk management |
| --- | --- | --- | --- | --- | --- | --- | --- | --- | --- | --- |
| Consumer complaints handling |  |  |  |  |  |  | No |  |  |  |

8. Credit risk management對其他Criteria的影響程度

| Criteria | Capital adequacy | Asset quality | Earnings | Supervising the board of directors and management level | Status of complying with major laws | Soundness of operation control | Consumer complaints handling | Credit risk management | Market risk management | Operational risk management |
| --- | --- | --- | --- | --- | --- | --- | --- | --- | --- | --- |
| Credit risk management |  |  |  |  |  |  |  | No |  |  |

9. Market risk management對其他Criteria的影響程度

| Criteria | Capital adequacy | Asset quality | Earnings | Supervising the board of directors and management level | Status of complying with major laws | Soundness of operation control | Consumer complaints handling | Credit risk management | Market risk management | Operational risk management |
| --- | --- | --- | --- | --- | --- | --- | --- | --- | --- | --- |
| Market risk management |  |  |  |  |  |  |  |  | No |  |

10. Operational risk management對其他Criteria的影響程度

| Criteria | Capital adequacy | Asset quality | Earnings | Supervising the board of directors and management level | Status of complying with major laws | Soundness of operation control | Consumer complaints handling | Credit risk management | Market risk management | Operational risk management |
| --- | --- | --- | --- | --- | --- | --- | --- | --- | --- | --- |
| Operational risk management |  |  |  |  |  |  |  |  |  | No |

~謝謝您的填答~
